# Testing the validity and reliability of the Arabic version of the painDETECT questionnaire in the assessment of neuropathic pain

**DOI:** 10.1371/journal.pone.0194358

**Published:** 2018-04-23

**Authors:** Amani Abu-Shaheen, Shehu Yousef, Muhammad Riaz, Abdullah Nofal, Isamme AlFayyad, Sarfaraz Khan, Humariya Heena

**Affiliations:** 1 Research Center, King Fahad Medical City, Riyadh, Saudi Arabia; 2 Anesthesia department, King Fahad Medical City, Riyadh, Saudi Arabia; 3 Epidemiology Department of Health Sciences, University of Leicester, Centre for Medicine, Leicester, United Kingdom; 4 King Saud University Medical City, Riyadh, Saudi Arabia; Weill Cornell Medicine-Qatar, QATAR

## Abstract

**Introduction:**

Neuropathic pain (NP) can cause substantial suffering and, therefore, it must be diagnosed and treated promptly. Diagnosis of NP can be difficult and if made by an expert pain physician is considered the gold standard, however where expert help may not be easily available, screening tools for NP can be used. The painDETECT questionnaire (PD-Q) is a simple screening tool and has been widely used in several languages. We developed an Arabic version of PD-Q and tested its validity and reliability.

**Methods:**

The original PD-Q was translated into the Arabic language by a team of experts. The translated version of the PD-Q was administered to the study population, which included patients having moderate to severe pain for at least three months. Reliability of the Arabic version was evaluated by an intra-class-correlation coefficient (ICC) between pre- and post-measures and Cronbach’s α values. Validity was measured by receiver operating characteristic (ROC) curve. Expert pain physician diagnosis was considered as the gold standard for comparing the diagnostic accuracy.

**Results:**

A total of 375 patients were included in the study, of which 153 (40.8%) patients were diagnosed with NP and 222 [59.2%] patients had nociceptive pain. The ICC between pre- and post-PD-Q scale total scores for the overall sample, NP group, and NocP group was 0.970 (95% CI, 0.964–0.976), 0.963 (95% CI, 0.949–0.973), and 0.962 (95% CI, 0.951–0.971), respectively. The Cronbach’s α values for the post-assessment measures in the overall sample, NP group, and nociceptive pain group, were 0.764, 0.684, and 0.746, respectively. The area under the ROC curve was 0.775 (95% CI, 0.725–0.825) for the PD-Q total score.

**Conclusion:**

The Arabic version of the PD-Q showed good reliability and validity in the detection of NP component in patients with chronic pain.

## Introduction

The International Association for the Study of Pain (IASP) defines pain as “an unpleasant sensory and emotional experience associated with actual or potential tissue damage or described regarding such damage” [1]. The two main types of pain, neuropathic pain (NP) and nociceptive pain (NocP), differ in their pathology and clinical presentation. While NP is caused by activation of nociceptors as a result of direct damage to tissues, NocP occurs due to disease or lesion of the somatosensory nervous system [2, 3]. Thus, the pain sensation in NP is without any apparent external injury [4]. Clinically, NP can present with burning type continuous pain, electric shock-like sensation, and mechanical allodynia. It is also characterized by hyperalgesia, paresthesia, and dysesthesia [5]. NP component is usually found in conditions such as back pain, diabetic polyneuropathy, post herpetic neuralgia, etc. [6, 7]. The pain is chronic and can lead to substantial suffering by causing anxiety, depression, sleep deprivation, frequent visits to the hospital, and reduced quality of life [8, 9]

Moreover, NP does not respond to conventional analgesics such as paracetamol and non-steroidal anti-inflammatory drugs (NSAIDs). Instead, NP is treated with certain other agents such as tricyclic antidepressants (amitriptyline, imipramine, clomipramine), serotonin-norepinephrine reuptake inhibitors (duloxetine, venlafaxine), and antiepileptics (pregabalin, gabapentin) [10]. Diagnosis of NP can be challenging, and diagnostic inaccuracy may lead to inappropriate treatment. It is, therefore, of utmost importance that the type of pain is accurately diagnosed.

Reaching the precise diagnosis of NP may be difficult especially in the primary care setting. Based on the distinct features of NP that distinguish it from NocP, several screening tools have been developed to help in the diagnosis [11]. While evaluation by an expert pain physician is considered as a gold standard for diagnosis of NP, these tools can be very useful for initial screening. Screening tools commonly used include the Leeds Assessment of Neuropathic Symptoms and Signs (LANSS), the Neuropathic Pain Questionnaire (NPQ), painDETECT questionnaire (PD-Q), and ID-Pain [12–15].

The PD-Q was first used in the German population in the year 2004. Since then, it has become very popular and has been translated into several languages including Spanish, Swedish, Dutch, Turkish, Japanese, and Korean [16–21]. More than 300,000 patients worldwide have been assessed for NP using the PD-Q [4]. Although PD-Q has been widely used, Arabic translation has not been developed and validated.

Patient access to care is different rural areas as compared to urban areas and different for different socioeconomic statuses. Lack of trained personnel is also a burning issue. To date there are no, regional guidelines for NeuP management, which are largely dependent on the local settings. To raise awareness among healthcare practitioners about NeuP, and to provide them a simple tool for its diagnosis to alleviate the suffering of the patients afflicted with chronic pain due to neuropathological causes, in the Kingdom of Saudi Arabia, especially in the remote areas where expert medical help is lacking. To address this unmet need, we developed an Arabic version of PD-Q and tested its validity and reliability.

## Materials and methods

### Study population

Adult patients attending any of the three clinics: pain management e diabetes and orthopedics at a single tertiary hospital in Riyadh, King Fahad Medical City (KFMC) were included in the study. Patients with moderate to severe NP or NocP pain (scoring 5 or higher on a 0–10 numerical rating scale) for a minimum of three months were included. Patients with the pain of unknown origin, mixed-type pain, and diffuse pain which includes fibromyalgia syndrome, myofascial pain, complex regional pain syndrome, cancer pain, and headaches wereexcluded. Patients with substance abuse, chronic alcoholism, severe depression, and those incapable of understanding the questionnaire were excluded [22].

The study was approved by the Institutional Review Board at KFMC, Riyadh, Saudi Arabia. Written informed consent was obtained from the participants who met inclusion criteria and agreed to participate in the study.

### Recruitment

The study participants were randomly approached and invited to participate in this study by the research team over a period of one year from March 2016 to March 2017.

### Translation of the questionnaire

The adaptation procedure was monitored by an expert panel including two specialists in pain management, an expert in research methodology, an expert in clinical research, and an expert in linguistics. The international guidelines for cross-cultural adaptation of health questionnaires and diagnostic tests were followed. An internationally-accepted translation methodology was used by a well-established linguistic validation process [23, 24].

Two bilingual translators, with Arabic mother tongue, translated the questionnaire to the Arabic language. While one of the translators was informed about the questionnaire and its applications, the other had no information about the questionnaire. Each translator independently produced a translated version. The two versions were then combined into a single questionnaire. This was followed by backward translation to English by two native English speakers who had no knowledge of the original version. A meeting was then held between the experts and the two translator teams to discuss the original and translated versions. The expert committee consolidated all the versions and developed the pre-final version for pre-testing. The pre-final version was administered to a sample of 30 patients as a part of pre-testing. Each patient was asked to fill-in the form and was then interviewed about his/her understanding of each item of the questionnaire and the corresponding response.

### Study design

The study population was divided into two groups. The first group included patients diagnosed with NP by a pain specialist in pain clinics as per the guidelines established by the IASP [3]. The second study group included patients with NocP.

The same investigator administered the Arabic version of the PD-Q to the study population twice (pre-clinic visit and post clinic visit) within a gap of two to four hours (S1 Arabic version of the painDETECT questionnaire).

### Description of PD-Q

The PD-Q is simple, self-administered, useful screening questionnaire that allows detecting NP component in patients with chronic pain. The PD-Q consists of four main sections. The first section contains three items with 11-point Likert scale format with anchor terms in the scale ends (0 = no pain, 10 = maximum pain), accompanied by a color grading scale representing pain intensity in analog format. These items assess the intensity of pain at the time of administration of the scale, and maximum pain intensity and average pain during preceding 4 weeks. The first section is used to diagnose the presence of pain and is not included in the questionnaire scoring. In the second section, patients are asked to mark one of the four graphs that best describe their pain course patterns. The possible patterns and their scores are determined as follows: persistent pain with slight fluctuations (0 points), persistent pain with pain attacks (−1 point), pain attacks without pain between them (1 point), and pain attacks with pain between them (1 point). The third section includes a sensory map representing homunculus along with questions designed to mark the pain zone, a dichotomous item about the presence of radiating pain and showing the direction of radiating pain with an arrow. The positive answer about the presence of radiating pain is scored with two points. In the last section, there are seven Likert type items asking about the intensity of the sensation marked over the homunculus. These items are scored with a 6-point Likert format, with corresponding ordinal anchor terms (0 = never, 1 = hardly noticed, 2 = slightly, 3 = moderately, 4 = strongly, 5 = very strongly). These Likert-type items enquire about the following sensations: burning, tingling or prickling, allodynia, pain attacks, temperature evoked pain, numbness, and pressure-evoked pain. This last section provides scores between 0 and 35 points. The final score is obtained summing up the scores of the last three sections with a total score of −1 to 38. Two cutoff values are used by the developer of PD-Q for the presence of NP. Scores ≤12 state that an NP component is unlikely and scores ≤19 indicate that neuropathic component is very likely to be present. Scores between 12 and 19 suggest that the result is unclear [15].

### Sample size estimation

The sample size was calculated assuming a significance level of α = 0.05 and prevalence of 41% with power = 0.95 and maximum difference = 0.5. The sample size that is needed to validate the questionnaire for the two groups was 376 patients.

### Statistical analysis

Socio-demographic and clinical characteristics of patients were summarized for the whole sample (NP group and NocP group) using frequency (%) for categorical variables and mean (standard deviation, SD) for continuous variables. For continuous variables, normality was assessed using Shapiro Wilk test and histograms. The socio-demographic and clinical characteristics were compared between the two groups using a chi-square test for categorical variables and t-test or Mann Whitney’s U test for continuous variables.

#### Examining reliability of PD-Q

The PD-Q responses were collected in two sessions, one pre-clinic visit and the other post- clinic visit. For the test re-test reliability of the items of PD-Q and its total scale score, intra-class-correlation coefficient (ICC) between pre- and post-measures were computed. For the pre- and post- clinic visits, correlation of the items of PD-Q with the total scale scores were assessed using Spearman correlation coefficients. Cronbach’s α for the pre- and post-measures of the items of PD-Q were computed to examine the internal consistency. All the above analyses were performed for the whole sample and also separately for the two study groups.

#### Assessing validity of PD-Q

Initially, median (interquartile range) of the total scores for PD-Q were computed and Mann Whitney’s U test was used to compare the scores between NP and NocP groups. Thereafter, to assess the discriminant validity of PD-Q in identifying the NP, we used receiver operating characteristics curve (ROC) analyses. A binary variable with a physician diagnosis of (NP = 1) versus (NocP = 0) was created. Physician diagnosis of neuropathic pain was carried out before the questionnaire was completed by the participants and the physicians were blinded to the responses on the questionnaires. In the logistic regression analysis, we used the physician diagnosis of neuropathic pain (a binary dependent variable) to assess how the total score of PD-Q scale predicts the physician diagnosis. This analysis assessed the validity of PD-Q by establishinga corroboration between physician diagnosis and questionnaire diagnosis for. Next, ith th binary variable, a ROC curve analyss was conducted for the total scale scores of PD-Q. concerning for each cut-off-point of the scores, sensitivity, specificity, correctly classified, positive likelihood ratio, negative likelihood ratio, was also calculated. In this analysis, using Youden’ s index [25], the best cut-off value of the scales total scores were determined. For the cut-off-value, the area under the ROC curve (95% CI) was computed, which determined the discriminant validity (diagnostic ability) of the total scores. All these analyses were separately performed for the pre and post-measure of the scales.

A statistical significance level of p<0.05 was used to reject the null hypothesis. The analyses were conducted using statistical software of SPSS software version 17.0 (Chicago, IL, USA) and Stata version 12 (StataCorp, Texas USA).

## Results

A total of 375 patients were included in the study. Expert pain physicians diagnosed NP in 153 (40.8%) patients and NocP in 222 (59.2%) patients. The mean age of patients in the NP and NocP groups was similar; however, the range was broader in patients with NocP (NP: 48.4±12.0 years [range, 21–71 years]; NocP: 48.9±14.5 years [range, 18–87 years]). Use of medication, diabetes, and heart disease were significantly more common in patients with NP. The duration of pain was more in patients with NP (Table 1).

**Table 1:** Demographic and clinical characteristics of patients in neuropathic and nociceptive pain groups.

Variables	Neuropathic pain(N = 153)	Nociceptive pain(N = 222)	p-value
**Male**, n (%)	51 (33.3)	58 (26.1)	0.131
**Education Level**, n (%)	77 (50.3)	133 (59.9)	
High school and above			0.066
**Occupation**, n (%)			
Employed vs. Unemployed	49 (32.0)	46 (20.7)	**0.016**
**Marital Status**, n (%)			
Married	136 (88.9)	176 (79.3)	**0.012**
Single	13 (8.5)	24 (10.8)	
Widow/Divorced	4 (2.6)	22 (9.9)	
**Medication use**, n (%)	126 (82.4)	119 (53.6)	**<0.001**
**Diabetes mellitus**, n (%)	60 (39.2)	65 (29.3)	**0.045**
**Hypertension**, n (%)	54 (35.3)	62 (27.9)	0.129
**Liver disease**, n (%)	6 (3.9)	3 (1.4)	0.168
**Heart disease**, n (%)	21 (13.7)	15 (6.8)	**0.024**
**Lung disease**, n (%)	8 (5.2)	6 (2.7)	0.205
**Kidney disease**, n (%)	8 (5.2)	10 (4.5)	0.747
**Physical exercise**, n (%)	44 (28.8)	41 (18.6)	**0.021**
**Age (years)**, mean (SD)	48.4 (12.0)	48.9 (14.5)	0.721
**Height (cm)**, mean (SD)	160.5 (10.3)	158.3 (9.2)	**0.045**
**Weight (kg)**, mean (SD)	82.0 (17.1)	78.9 (18.5)	0.138
**Body mass index (kg/m^2^)**, mean (SD)	32.0 (7.1)	31.6 (7.7)	0.665
**Duration of pain (month)**, mean [Min -Max]	48 [24–84]	24 [12–48]	**0.002**

Significant odds ratios (OR) are in bold.

Paracetamol was the most common painkiller used in both NP (n = 71; 46.4%) and NocP (n = 56; 25.2%) groups. Paracetamol was followed by meloxicam (NP: n = 35, 22.9%; NocP: n = 34, 15.3%), and diclofenac sodium (NP: n = 35; 22.9%; NocP: n = 40; 18.0%) in both the groups.

**Fig 1:**
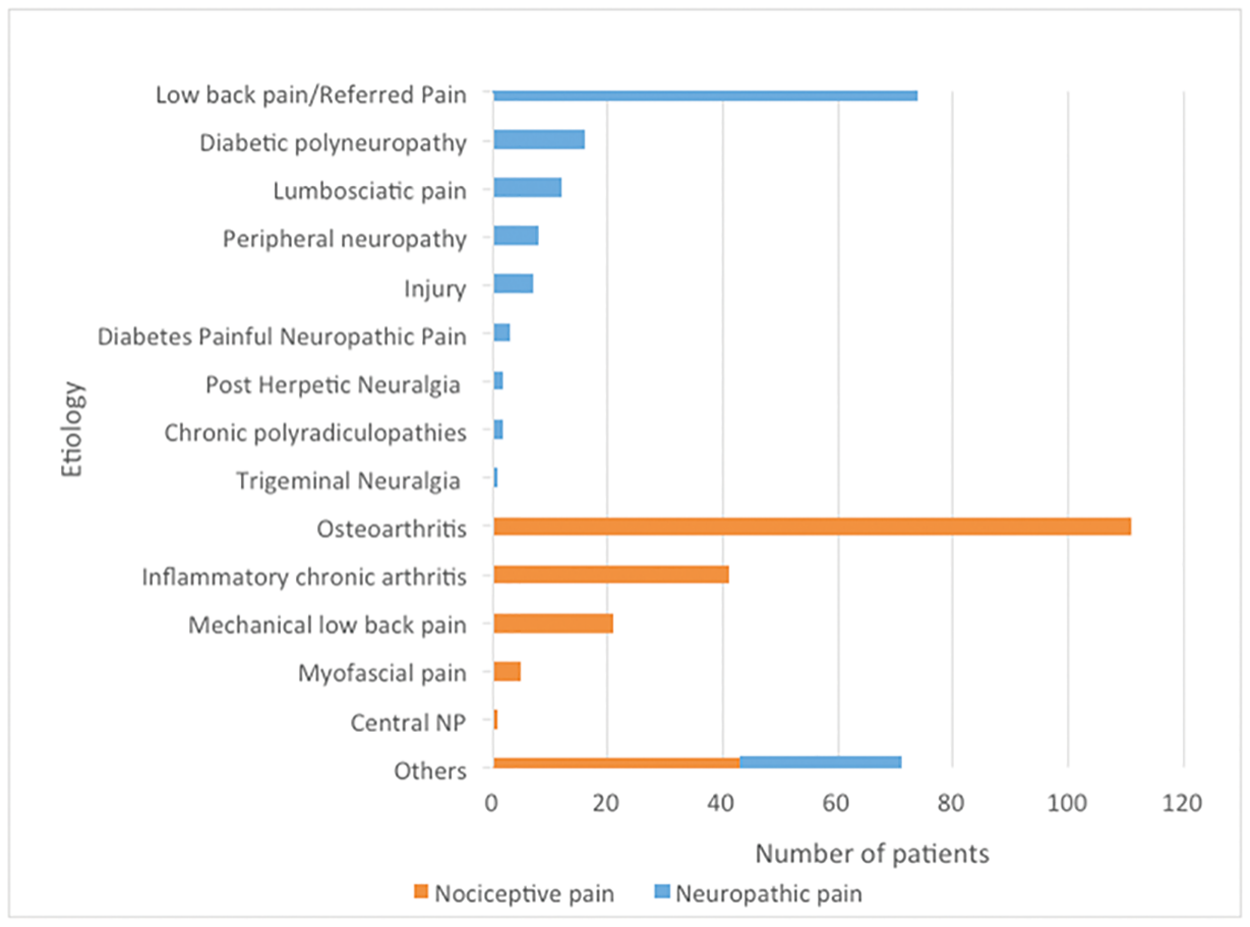
Etiology of pain in the two study groups.

NP was most commonly seen in patients with low back pain/referred pain (74 [48.4%]), and NocP was most commonly seen in patients with osteoarthritis (111 [50.0%]) (Fig 1).

### Reliability of the PD-Q scale

There was a high level of test-re-test reliability for the items of the PD-Q scale in the overall sample and the two study groups. The ICC between pre- and post-PD-Q scale total scores for the overall sample, NP group, and NocP group was 0.970 (95% CI, 0.964–0.976), 0.963 (95% CI, 0.949–0.973), and 0.962 (95% CI, 0.951–0.971), respectively. For the measures of the scale in the post clinic visit, the Cronbach’s α values were 0.764, 0.684, and 0.746, in the overall sample, in NP group, and in the NocP group, respectively. For the pre-clinic visit measures of the items of the PD-Q scale, a similar level of internal consistency was observed (Table 2).

**Table 2:** Test-re-test reliability: Agreement between pre- and post-clinic visits of PD-Q response and its internal consistency.

Items of the PD-Q	Overall sample		Neuropathic pain		Nociceptive pain	
	Agreement n (%)	ICC (95% CI)	Agreement n (%)	ICC (95% CI)	Agreement n (%)	ICC (95% CI)
How would you assess your pain now, at this moment?	85.6	0.971 (0.964–0.976)	83.0	0.973 (0.962–0.980)	87.4	0.969 (0.960–0.976)
How strong was the strongest pain during the past 4 weeks?	90.7	0.985 (0.982–0.988)	88.9	0.993 (0.991–0.995)	91.9	0.979 (0.972–0.984)
How strong was the pain during the past 4 weeks on average?	84.8	0.960 (0.951–0.967)	81.1	0.931 (0.905–0.950)	87.4	0.977 (0.969–0.982)
Mark the picture that best describes the course of your pain	93.3	0.909 (0.888–0.925)	92.2	0.905 (0.869–0.931)	94.1	0.913 (0.887–0.933)
Does your pain radiate to other regions of your body?	96.3	0.961 (0.952- 0.968)	98.0	0.965 (0.952–0.974)	95.1	0.945 (0.928–0.958)
Do you suffer from a burning sensation (e.g., stinging nettles) in the marked areas?	89.6	0.943(0.931–0.954)	85.0	0.942 (0.920–0.958)	92.8	0.933 (0.913–0.949)
Do you have a tingling or prickling sensation in the area of your pain (like crawling ants or electrical tingling)?	85.3	0.945 (0.933–0.955)	79.7	0.914 (0.882–0.937)	89.2	0.961 (0.949–0.970)
Is light touching (clothing, a blanket) in this area painful?	91.2	0.937 (0.922–0.948)	84.3	0.924 (0.896–0.945)	96.0	0.947 (0.931–0.959)
Do you have sudden pain attacks in the area of your pain, like electric shocks?	89.9	0.958 (0.948–0.965)	88.2	0.966 (0.954–0.976)	91.0	0.940 (0.921–0.954)
Is cold or heat (bath water) in this area occasionally painful?	86.9	0.920 (0.902–0.934)	79.1	0.898 (0.860–0.926)	92.3	0.936 (0.917–0.951)
Do you suffer from a sensation of numbness in the areas that you marked?	84.8	0.948 (0.936–0.957)	80.4	0.919 (0.888–0.941)	87.8	0.953 (0.939–0.964)
Does slight pressure in this area, e.g., with a finger, trigger pain	89.0	0.957 (0.947–0.965)	87.6	0.955 (0.938–0.967)	90.1	0.959 (0.947–0.969)
Total scale score:	-	0.970 (0.964–0.976)	-	0.963 (0.949–0.973)	-	0.962 (0.951–0.971)
**Consistency** of the above 7 items of pain scale	**Cronbach’s α**		**Cronbach’s α**		**Cronbach’s α**	
Pre-clinic visit	0.575		0.518		0.505	
Post-clinic visit	0.614		0.528		0.536	
**Consistency** of all 12 items of the PD-Q						
Pre-clinic visit	0.746		0.673		0.737	
Post-clinic visit	0.764		0.684		0.746	

**Agreement:** Observed agreement (%) of the items of **PD-Q** between pre- and post-clinic visits.

**ICC (95% CI):** Intra class correlation coefficient (95% confidence interval) for examining test re-test reliability of each items of the PD-Q scale and its total score (continuous variables).

**Cronbach’s α (alpha):** Coefficient used to examine internal consistency of the **PD-Q** scale.

The total scores for both pre-clinic visit and post clinic visit on the fourth section of the PD-Q scale, which includes seven items were evaluated using the Spearman rank correlation co-efficient, a significant (moderate to high) correlation between the total score and each item of the scale was observed (Table 3).

**Table 3:** Spearman rank correlation co-efficient total score for PD-Q scale with each of its items.

	Total score for PD-Q scale					
Items of PD-Q scale	Pre-assessment			Post-assessment		
	Overall sample (n = 375)	Neuropathic pain (n = 153)	Nociceptive pain (n = 222)	Overall sample (n = 375)	Neuropathic pain (n = 153)	Nociceptive pain (n = 222)
Do you suffer from a burning sensation (e.g., stinging nettles) in the marked areas?	0.592	0.532	0.551	0.618	0.552	0.537
Do you have a tingling or prickling sensation in the area of your pain (like crawling ants or electrical tingling)?	0.493	0.610	0.409	0.494	0.574	0.408
Is light touching (clothing, a blanket) in this area painful?	0.465	0.437	0.432	0.496	0.456	0.449
Do you have sudden pain attacks in the area of your pain, like electric shocks?	0.636	0.596	0.579	0.642	0.592	0.580
Is cold or heat (bath water) in this area occasionally painful?	0.444	0.395	0.434	0.465	0.407	0.421
Do you suffer from a sensation of numbness in the areas that you marked?	0.620	0.446	0.561	0.638	0.455	0.560
Does slight pressure in this area, e.g., with a finger, trigger pain	0.334	0.443	0.333	0.354	0.445	0.374

All correlations coefficients were statistically significant at p<0.001

### Validity of the PD-Q scale

**Fig 2:**
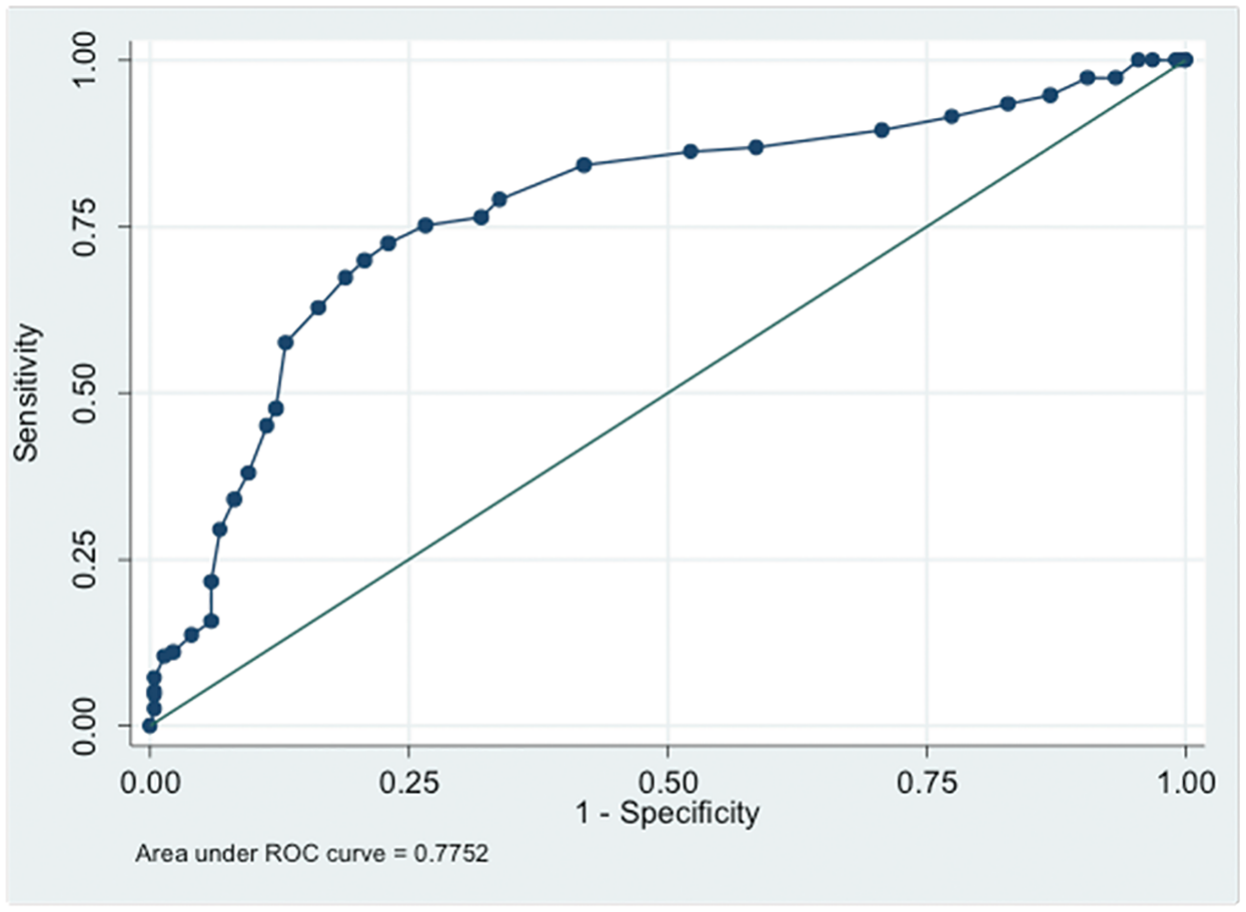
ROC curve analysis: Plot of sensitivity versus 1-specificity (PD-Q scale).

Considering the diagnosis as NP or NocP by a pain physician as the gold standard, when the discriminant validity was examined using the receiving operating characteristics (ROC) curve analyses, the area under the ROC curve was estimated to be 0.775 (95% CI, 0.725–0.825) for the PD-Q total score (Fig 2).

The adjusted odds ratios of the clinical diagnosis of neuropathic pain for per unit increase in the total score PD-Q scale was 1.15 (95% CI, 1.10–1.20; *P*<0.001) (Table 4). The final score is obtained summing up the scores of the last three sections with a total score of −1 to 38. Two cutoff values are used by the developer of PD-Q for the presence of NP. Scores ≤12 state that a NP component is unlikely and scores ≤19 indicate that neuropathic component is very likely to be present. Scores between 12 and 19 suggest that the result is unclear. The area under the ROC curve was 0.775 (95% CI, 0.725–0.825) for the PD-Q total score. Further discriminant statistics (i.e., percent sensitivity, specificity, correct classification, positive and negative likelihood ratio) for each of the possible cut-off values are presented in Table 5. For example, for the cut-off value of total score ≥ 16, the sensitivity was 75.2%, specificity was 73.4%, the correct classification was 74.1%, positive likelihood ratio was 2.828, and negative likelihood ratio was 0.338.

**Table 4:** Logistic regression models to adjust the effect of total scale score in predicting the physician diagnosis of neuropathic pain.

Variables	PD-Q scale	
	OR (95% CI)	p-values
**Total scores**	1.15 (1.10, 1.20)	<0.001
Employed vs. Unemployed	2.44 (1.24, 4.79)	0.009
Marital Status	0.47 (0.28, 0.82)	0.007
Medication use	0.30 (0.16, 0.57)	0.000
Diabetes mellitus	0.72 (0.39, 1.35)	0.306
Heart disease	0.54 (0.21, 1.42)	0.214
Physical exercise	0.37 (0.19, 0.73)	0.004
Height (cm)	1.03 (1.00, 1.06)	0.070
Duration of Pain (month)	1.01 (1.00, 1.01)	0.115

**Table 5:** Discriminative characteristics of the total score for PD-Q scale in identifying patients with neuropathic pain versus nociceptive pain (ROC curve analysis).

Cut point	Sensitivity (%)	Specificity (%)	Correctly classified (%)	LR+	LR-
(≥ -1)	100.0	0.0	40.8	1.000	
(≥ 0)	100.0	0.5	41.1	1.005	0.000
(≥ 2)	100.0	0.9	41.3	1.009	0.000
(≥ 3)	100.0	3.2	42.7	1.033	0.000
(≥ 4)	100.0	4.5	43.5	1.047	0.000
(≥ 5)	97.4	6.8	43.7	1.044	0.387
(≥ 6)	97.4	9.5	45.3	1.076	0.276
(≥ 7)	94.8	13.1	46.4	1.090	0.400
(≥ 8)	93.5	17.1	48.3	1.128	0.382
(≥ 9)	91.5	22.5	50.7	1.181	0.377
(≥ 10)	89.5	29.3	53.9	1.266	0.357
(≥ 11)	86.9	41.4	60.0	1.485	0.315
(≥ 12)	86.3	47.8	63.5	1.651	0.288
(≥ 13)	84.3	58.1	68.8	2.013	0.270
(≥ 14)	79.1	66.2	71.5	2.341	0.316
(≥ 15)	76.5	68.0	71.5	2.391	0.346
(≥ 16)	75.2	73.4	74.1	2.828	0.338
(≥ 17)	72.6	77.0	75.2	3.158	0.356
(≥ 18)	69.9	79.3	75.5	3.375	0.379
(≥ 19)	67.3	81.1	75.5	3.558	0.403
(≥ 20)	62.8	83.8	75.2	3.869	0.445
(≥ 21)	57.5	86.9	74.9	4.403	0.489
(≥ 22)	47.7	87.8	71.5	3.923	0.595
(≥ 23)	45.1	88.7	70.9	4.005	0.619
(≥ 24)	37.9	90.5	69.1	4.008	0.686
(≥ 25)	34.0	91.9	68.3	4.192	0.718
(≥ 26)	29.4	93.2	67.2	4.353	0.757
(≥ 27)	21.6	94.1	64.5	3.683	0.833
(≥ 28)	15.7	94.1	62.1	2.679	0.896
(≥ 29)	13.7	96.0	62.4	3.386	0.899
(≥ 30)	11.1	97.8	62.4	4.933	0.909
(≥ 31)	10.5	98.7	62.7	7.739	0.908
(≥ 32)	7.2	99.6	61.9	15.961	0.932
(≥ 33)	5.2	99.6	61.1	11.608	0.952
(≥ 34)	4.6	99.6	60.8	10.157	0.959
(≥ 36)	2.6	99.6	60.0	5.804	0.978
(> 36)	0.0	100.0	59.2		1.000

Area under receiving operating characteristics (ROC) curve (95% CI) = 0.775 (0.725–0.825)

**LR+:** Positive likelihood ratio

**LR-:** Negative likelihood ratio

**Cut-point:** cut-off-value of the **PD-Q** total scale score.

## Discussion

In this study, the most common agent used for pain, irrespective of the pain type, was paracetamol followed by NSAIDs. This may mean that patients with NP either had no pain relief or had only partial relief, as they were not receiving appropriate treatment. If the diagnosis of NP is made correctly early on, appropriate treatment can be instituted at primary care health facility and patients can benefit from it. A pilot study conducted by Hassan et al. from 10 centers in the Middle East Region in 2004 puts the prevalence of NP as 41% in the chronic low back pain patients and NocP as 59% [26]. In a prospective, multicenter, epidemiological study was conducted to assess the prevalence of NP among adults with lower back pain in the Arabian Gulf region. Out of 1134 patients, 628 (55%) patients were classified as having NP [27].

To achieve an accurate diagnosis, assessment by an expert pain physician including meticulous history, clinical examination, and neurological evaluation must be carried out [28, 29]. It has been suggested that the diagnosis of NP component is missed in a substantial number of patients with pain [30]. In resource-challenged settings like in KSA where there is a lack of expertise in primary care level due to non-availability of avenues for in-service training and skill expansion of health care providers [31], screening tools such as the PD-Q can prove to be extremely useful. The strength of PD-Q also lies in its simplicity and ease of use. This tool is very convenient and can be easily administered by anyone without having to rely on a pain expert.

The original version of the PD-Q, which was evaluated in a large population of about 8000 subjects, demonstrated high sensitivity, specificity, and positive predictive accuracy. This screening tool can be used in primary care settings worldwide only if it is translated and adapted for local use and tested for psychometric properties. Several translations of the PD-Q have been developed and assessed in the corresponding local populations [16–21].

In this study, the first important step was a translation of the original version to the Arabic language. The translation which was done as per the standard textbook Arabic language understandable by most of the Arabic speakers and was not limited to the Saudi dialect only. A panel of experts was employed to ensure that the translation and adaptation was done as per internationally accepted norms. Additionally, the prefinal version was tested on a sample population before administering it to the study population. Expert pain physicians were involved at several stages.

The demographics of both study groups including age, gender, occupation, and level of education were fairly balanced indicating that there was no difference in the level of understanding of the questionnaires between the groups. The Arabic version of the PD-Q showed good psychometric properties. Stability of the PD-Q over time (i.e., the test-retest reliability) was excellent. The test-retest reliability was similar to previous studies [16, 17]. The ICC value of the Spanish version was reported as similar to that of the Turkish version [16, 17]. Further, internal consistency achieved for the total score in this study (Cronbach’s α = 0.764) was close to the value attained by the Turkish version (Cronbach’s α = 0.81) of the PD-Q [17], and also comparable to the original (Cronbach’s α = 0.83) and Spanish versions (Cronbach’s α = 0.86) [15, 16]. In this study, when the internal consistency of all the 12 items of the PD-Q scale was assessed, it was in an acceptable range. The discriminant validity of the Arabic PD-Q version was also good for NP versus NocP. Furthermore, the results of sensitivity, specificity, and correct classification for NP versus NocP were similar to those obtained in other studies [15, 16].

Hence, the Arabic version of PD-Q is reliable and valid scale for detecting NP in the Arabic population. Especially in remote areas where expert help may not be available, the PD-Q can be a real boon. It can serve as a powerful tool for initial screening of patients with pain.

Our study also had some limitations. This was a single tertiary hospital study as patients from multiple centers could not be included due to logistic reasons.

## Conclusion

In summary, we developed the Arabic version of the PD-Q and tested its psychometric properties. The Arabic version of PD-Q demonstrated good reliability and validity. A large-scale study in the Arabic population is required to confirm the results of this study further and further affirm the validity and reliability of the Arabic version of the PD-Q.

## Supporting information

S1 Arabic version of the painDETECT questionnaire (PUB)
